# Ethylene Responsive Factor MeERF72 Negatively Regulates *Sucrose synthase 1* Gene in Cassava

**DOI:** 10.3390/ijms19051281

**Published:** 2018-04-25

**Authors:** Chen Liu, Xin Chen, Ping’an Ma, Shengkui Zhang, Changying Zeng, Xingyu Jiang, Wenquan Wang

**Affiliations:** 1 Institute of Tropical Agriculture and Forestry, Hainan University, Haikou 570228, China; liuchenneo@163.com; 2 The Institute of Tropical Bioscience and Biotechnology, Chinese Academy of Tropical Agricultural Sciences, Haikou 571101, China; chenxin@itbb.org.cn (X.C.); zsk8920@gmail.com (S.Z.); zengchangying@itbb.org.cn (C.Z.); 3 Key Laboratory of Biology and Genetic Resources of Tropical Crops, Ministry of Agriculture, Haikou 571101, China; 4 College of Biological Engineering, Henan University of Technology, Zhengzhou 450001, China; pinganma@163.com; 5 College of Plant Science & Technology, Huazhong Agricultural University, Wuhan 430070, China

**Keywords:** cassava, starch, sucrose synthase, gene regulation, transcription factor

## Abstract

Cassava, an important food and industrial crop globally, is characterized by its powerful starch accumulation in its storage root. However, the underlying molecular mechanism for this feature remains unclear. Sucrose synthase initializes the conversion of sucrose to starch, and, to a certain extent, its enzyme activity can represent sink strength. To understand the modulation of *MeSus* gene family, the relatively high expressed member in storage root, *MeSus1*, its promoter was used as bait to screen cassava storage root full-length cDNA library through a yeast one-hybrid system. An ethylene responsive factor cDNA, designated as *MeERF72* according to its homolog in *Arabidopsis*, was screened out. The transcript level of *MeERF72* was induced by ethylene, drought, and salt treatments and repressed by abscisic acid, Auxin, gibberellin, salicylic acid, and low and high temperatures. The MeERF72 protein has a conserved APETALA2 domain in its N-terminus and an activated domain of 30 amino acids in its C-terminus, can bind to *MeSus1* promoter in vitro and in vivo, and represses the promoter activity of *MeSus1*. MeERF72 is a transcription factor that can negatively regulate the expression level of *MeSus1* in cassava.

## 1. Introduction

Cassava (*Manihot esculenta* Crantz) ranks in the top six food crops in the world, especially in the tropics, because of its high efficient accumulation of carbohydrates in its storage roots while also adaptable to the high luminous intensities and high temperatures of tropical environments. In the starch biosynthesis pathway, sucrose synthase (Sus), in the first step, catalyzes the reciprocally exploded sucrose into uridine diphosphate glucose (UDPGlc). Also, UDPGlc is the precursor of starch synthesis. The Sus enzyme activity can be induced by sucrose and oxygen deficiency in wound treatments [1]. Sus is present in various plant tissues and shows higher enzyme activity in sink organs.

Overexpressing or silencing a specific member of the *Sus* gene family in plants could significantly affect their phenotype. Repression of *Sus* expression in potato to 2–6% of the wild type results in the decrease in starch content and root yield [2]. Meanwhile, *Sus4* overexpression led to the improvement of starch, adenonsine diphosphate (ADPG), UDPGlc content, and root/seed yield in potato and maize [3,4]. Furthermore, *Sus* overexpression from *Arabidopsis* and *Populus* in tobacco increased cell sucrose content, cell wall thickness, plant height and shortened life cycle [5,6]. The enzyme activity of *Sus* significantly affects the ability to assimilate photosynthetic products in plant sink organs, indicating that it is an important enzyme for starch biosynthesis, and its activity can represent sink strength in plant.

Ethylene is a gaseous plant hormone widely involved in the regulation of plant development and senescence, particularly seed germination, seedling growth, flowering, organ abscission and fruit maturation [7], and ethylene inhibitors could enhance sucrose synthase activity and promote the grain filling of basal rice kernel [8]. It is also a signal to adapt to environmental changes via an ethylene–ETRs (ethylene resistant)–CTR1 (constructive triple response)–EIN2 (ethylene insensitive 2)–EIN3/EILs (ethylene insensitive 3/EIN3 like 1)–ERFs (ethylene responsive factors)–ethylene response [9,10]. ERFs belong to the APETALA2/ethylene response factors (AP2/ERF) subfamily and are located in the most downstream of the transduction pathway [11]. Previous reports demonstrated that ERFs are involved in the regulation of starch biosynthesis genes aside from its already known functions. For instance, *OsRSR1*, a member of AP2/ERF subfamily, negatively co-expressed with starch biosynthesis related genes, and *rsr1* mutants have larger seed size, higher starch content and yield [12]. OsSERF72 also can negatively regulate the expression levels of *GBSSI*, *SSI*, *SSIIIa*, and *AGPL2* and directly bind to the promoter of *GBSSI* [13]. ERFs appeared to be negative regulators for starch synthesis, but whether ERFs have similar function in cassava starch synthesis is unknown.

Here, we report an *ERF* gene from cassava, named *MeERF72*, according to its homolog in the *Arabidopsis* ERF gene family. The MeERF72 protein can bind to the promoter of *MeSus1* gene via a yeast one-hybrid assay in vivo and DNA-Protein-Interaction assay in vitro. MeERF72 is a negative regulator of *MeSus1* and is involved in cassava carbohydrate fixation.

## 2. Results

### 2.1. Full-Length cDNA Library for Y1H Screening

Full-length double-strand complementary DNA were synthesized using the mRNA of cassava storage roots as templates, then together with linearized pGADT7-Rec3 vectors were co-transformed into competent cells of yeast strain Y187. The double-strand cDNAs directionally linked to pGADT7-Rec3 vectors in the yeast cells through homologous recombination. Lastly, the total capacity of yeast library was 4.0 ± 1.7 × 10^6^ cfu. Subsequently, the recombined plasmid was isolated from yeast cells and then transformed into competent cells of *E. coli* strain Stellar. Its total capacity was 1.4 ± 0.3 × 10^7^ cfu with over 70% of the insertion of more than 750 bp (Figure S1). Given that the *ccdB* gene was introduced into pGADT7-Rec3 vector, all clones had recombinants because the strains that contained non-recombined pGADT7-Rec3 vectors did not survive. Generally, our cDNA library in yeast or *E. coli* can be easily stored and amplified, and is thus a reusable resource for Y1H and Y2H screening.

### 2.2. Cloning and Characterization of MeERF72

Sus initializes the conversion of sucrose to starch, and its enzyme activity can represent sink strength in plants. The cassava *Sus* gene family has seven members based on homologous basic local alignment search tool (BLAST) search wherein the amino acid sequences of *Arabidopsis* Sus family were used as queries. *MeSus1* has relatively high expression level in storage root compared with other tested tissues (Figure S2). To understand the regulatory mechanism of *MeSus1* in the storage root of cassava, we performed a Y1H assay with *MeSus1* promoter as bait and our storage root cDNA library as prey. Successfully, *MeERF72* and more than 20 other candidate transcription factors (TF) were screened out after re-checking, subsequent sequencing and functional annotation.

**Figure 1:**
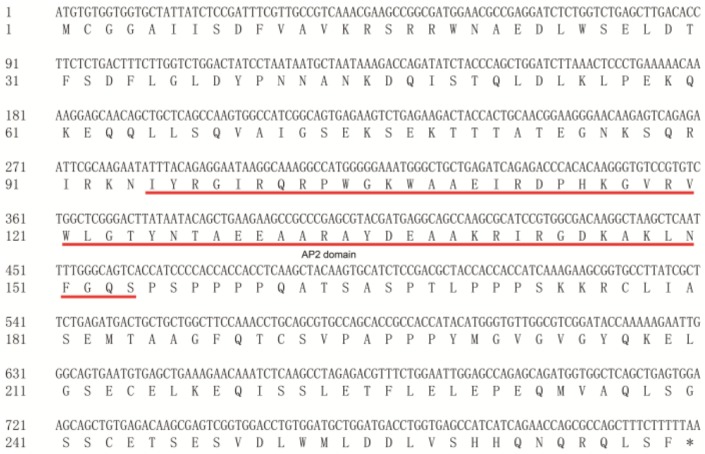
The conserved APETALA2 (AP2) DNA binding domain in the ERF72 protein. Conserved AP2 domain is indicated by underline. The stop code marks as *.

**Figure 2:**
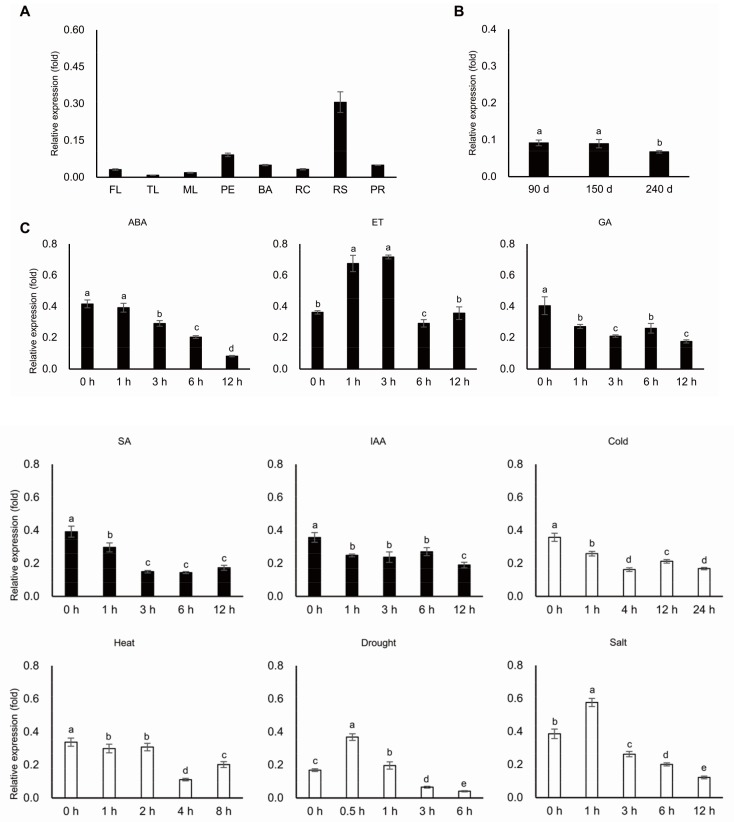
Transcription profiles of *MeERF72*. (**A**) Expression profile of *MeERF72* in different organs of cassava plant. F: flower, TL: tip leaf, ML: mature leaf, P: petiole, SR: stem rind, RC: root cortex, RS: root stele, PR: primal root; (**B**) Expression profile of *MeERF72* in three growth stages of storage root; d: day; (**C**) Transcription profiles of *MeERF72* respond to abscisic acid, ethylene, giberellin, salicylic acid, indole acetic acid, and low and high temperatures (42 °C), drought, salt. The Y axis is the scale of the relative expression levels. Error bars represent the SD of four technical replicates, the significant difference is assessed by analysis of variance (ANOVA) at *p* < 0.05.

MeERF72 (Manes.15G009900), encoded a protein of 269 amino acid residues with an AP2 DNA binding domain in the N-terminus region (Figure 1), identified by protein-protein BLAST program of PlantTFDB with the *E*-value of 5 × 10^−45^ to AtERF72 (At3g16770). MeERF72 was extremely higher expressed in the root stele than in other tissues of the cassava plant (Figure 2A), and its expression level was gradually decreased along with the storage root enlargement (Figure 2B). Ethylene treatment significantly up-regulated the transcript level of MeERF72, but abscisic acid, gibberellin, indole acetic acid, salicylic acid, and low temperature treatments obviously repressed its transcriptional level. In addition, drought and salt stresses induced its expression level (Figure 2C).

### 2.3. MeERF72 Binds to MeSus1 Promoter through AP2 Domain

**Figure 3:**
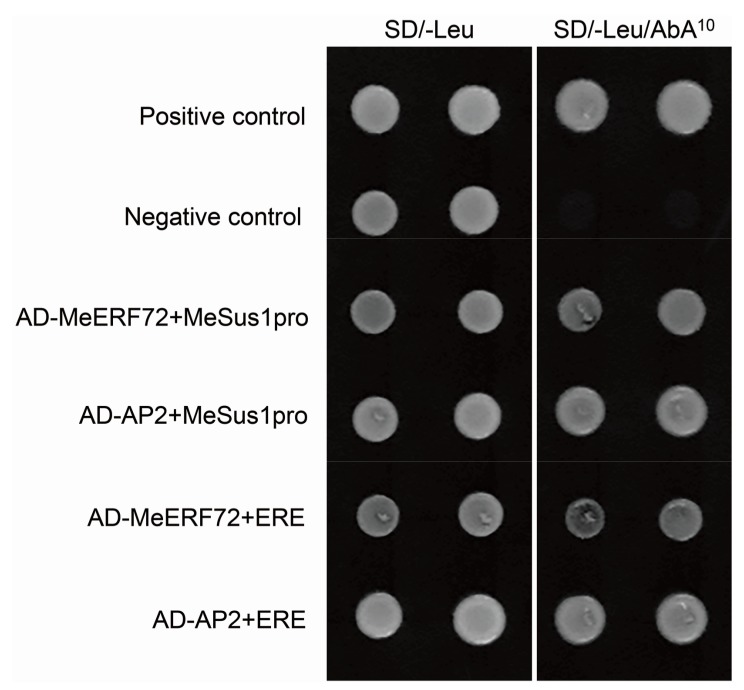
ERF72 protein/AP2 domain can bind to *MeSus1* promoter and its ethylene responsive element (ERE). Yeast cells that carried AD-53+p53-AbA (Positive control), AD-53+pAbAi (Negative control), AD-MeERF72+MeSus1pro-AbA, AD-AP2+MeSus1pro-AbA, AD-MeERF72+pERE-AbA and AD-AP2+pERE-AbA were grown in SD/-Leu selective medium containing 10 ng/mL AbA for three days at 30 °C.

The full-length CDS of *MeERF72* was cloned, and an AP2 DNA binding domain was found in the MeERF72 protein. Thus, two prey vectors, pGADT7-MeERF72 and pGADT7-AP2, were transformed into two bait strains Y1HGold/pSus1pro-AbA and Y1HGold/pERE1-AbA, respectively. The results showed that all four transformed yeast cells grew in the SD/-Trp/AbA^10^ medium, i.e., AP2 interacted with ERE1 (Figure 3), indicating that the MeERF72 protein can bind to the promoter of *MeSus1* and the AP2 domain is indeed the DNA binding domain, and its accurate binding site may be the ERE (TTTGAAAT), which is located in the region from −1384 to −1376 bp of the *MeSus1* promoter.

### 2.4. The Activated Domain Is Located in the aa212-aa241 Region of MeERF72 Protein

**Figure 4:**
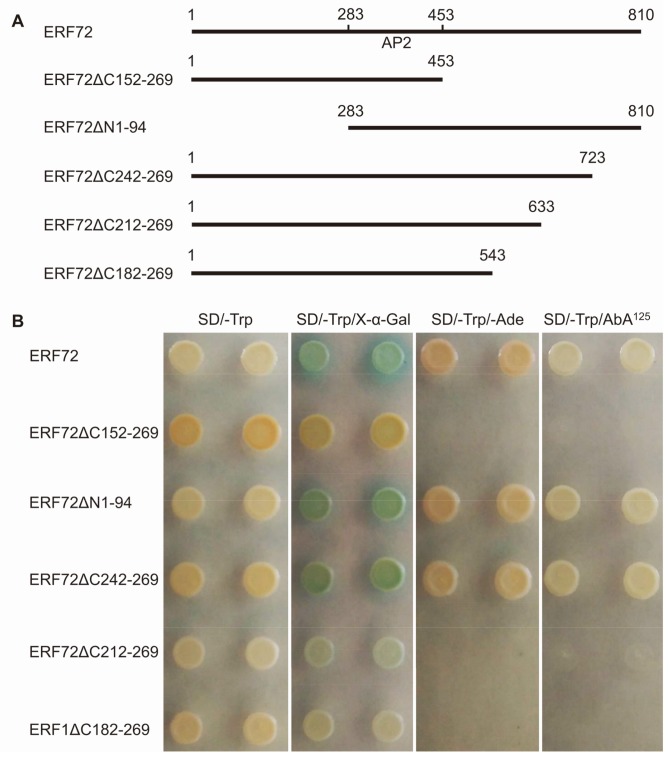
Region aa212-aa241 is the activated domain in MeERF72 protein. (**A**) Different deletion vectors of ERF72 protein: ERF72ΔC152-269 means that aa152 to aa269 is deleted from C-terminus, ERF72ΔN1-94 means that aa1-aa94 is deleted from N-terminus; (**B**) Activation of reporter genes by different deletions of ERF72 protein (SD/-Trp/AbA^125^ contains 125 ng/mL AbA).

To determine whether MeERF72 protein directly activates the downstream genes, we fused its full-length CDS to the GAL4 DNA binding domain in the pGBKT7 vector and then transformed the resulting complex into Y2HGold. The transformed cells, which contained pGBKT-MeERF72, survived in the SD/-Trp, SD/-Trp/-Ade, and SD/-Trp/AbA^125^ media, and their colonies turned blue in the SD/-Trp/X-α-Gal medium (Figure 4). This result indicated that MeERF72 had trans-activation ability in the yeast cells. Two sub-CDS of *MeERF72*, ERF72ΔC152-269 and ERF72ΔN1-94 were fused to the pGBKT7 vector and transformed into yeast cells. The results showed that ERF72ΔN1-94 had trans-activation ability. Furthermore, C-terminus truncated deletions, ERF72ΔC242-269, ERF72ΔC212-269, and ERF72ΔC182-269, were transformed into yeast cells. Only ERF72ΔC242-269 activated the reporter genes. In summary, aa212 to aa241 region is the putative activated domain of the MeERF72 protein.

### 2.5. MeERF72 Is a Nuclear-Localized Protein

**Figure 5:**
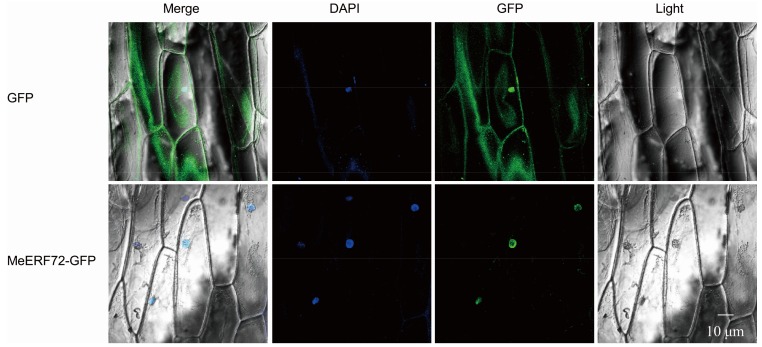
Nuclear localization of MeERF72. Top row/bottom row: the corresponding bright field, fluorescence, merged fluorescence image, and DAPI image of MeERF72-GFP/GFP control.

To ensure the sub-cellular location of MeERF72 protein, *MeERF72* gene was fused into the pSL1 plus vector (Figure S3A) and transiently expressed in onion epidermal cells via *Agrobacterium*-mediated impregnation, and was observed under a laser scanning confocal microscope. The MeERF72-GFP fusion protein was located in the nucleus of the onion epidermal cells, and the GFP was dispersed in the onion epidermal cell of wild type (Figure 5). This indicated that MeERF72 localized in the cell nucleus.

### 2.6. MeERF72 Can Bind to the MeSus1 Promoter In Vitro

**Figure 6:**
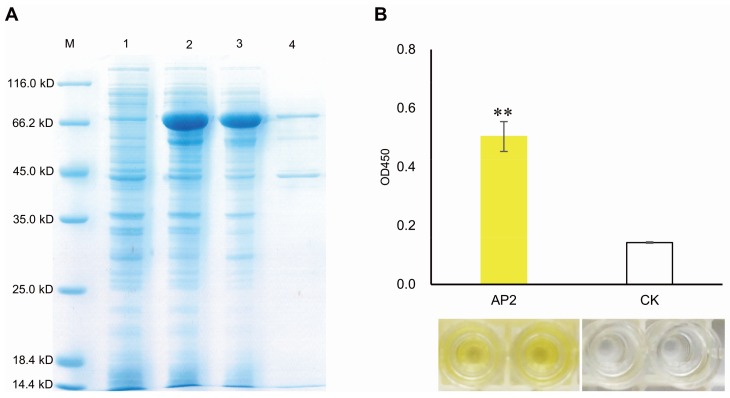
AP2 domain of MeERF72 binding to the promoter of *MeSus1* as analyzed DPI-ELISA. (**A**) Over-expression of MeERF72 in *E. coli*. M: molecular markers, (1) *E. coli* cells harboring pCold Trigger Factor-AP2 not induced; (2) *E. coli* cells harboring pCold Trigger Factor-AP2 after 20 h of induction; (3) the clear supernate of total protein from *E. coli* cells harboring pCold Trigger Factor-AP2 after 20 h of induction; (4) Purified Trigger Factor-AP2 fusion protein; (**B**) AP2 interacts with the *MeSus1* promoter by DPI-ELISA assay. AP2: double-strand biotinylated *MeSus1* promoter DNA probe + purified Trigger Factor-AP2 fusion protein; CK: double-strand biotinylated *MeSus1* promoter DNA probe + trigger factor. Error bars represent the SD of four technical replicates, the significant difference is assessed by ANOVA, ** *p* < 0.01.

To investigate whether MeERF72 binds to the *MeSus1* promoter in vitro, we extracted a purified recombinant Trigger Factor-AP2 protein, like an artificial TF, from *E. coli*, and its molecular mass matched to the predicted value (Trigger Factor 48.1 kD + AP2 11.1 kD = 59.2 kD, Figure 6A). Then, DPI-ELISA assay was carried out with double-strand biotinylated *MeSus1* promoter and recombinant Trigger Factor-AP2 protein. The results indicated that Trigger Factor-AP2 interacts with *MeSus1* promoter because the absorbance of Trigger Factor-AP2+*MeSus1* promoter was significantly higher than the control (Figure 6B). This shows that MeERF72 can bind to the *MeSus1* promoter in vitro.

### 2.7. Repression of MeSus1 Promoter Activity by MeERF72

**Figure 7:**
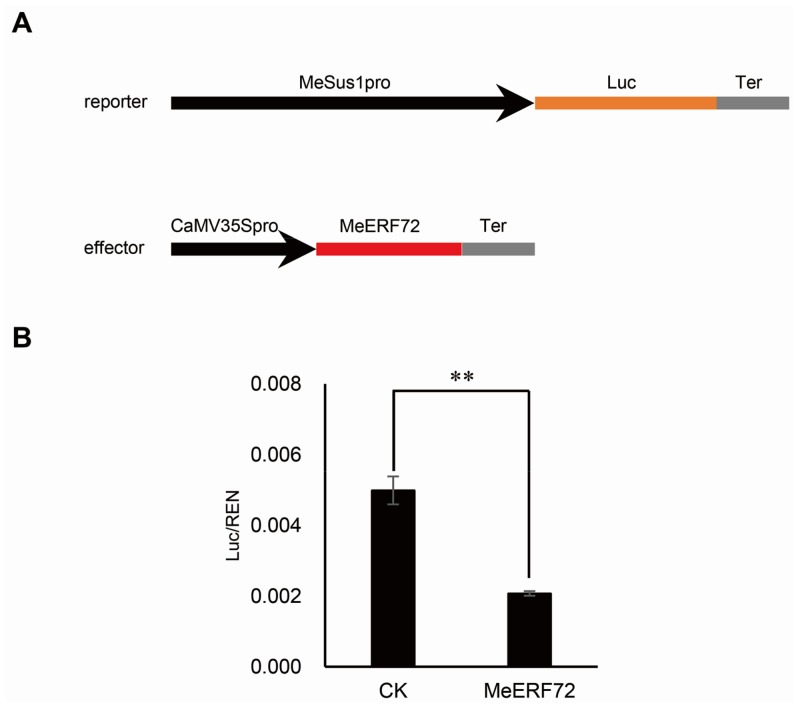
Repression of *MeSus1* promoter in the transient expression system by MeERF72. (**A**) Reporter and effector vector sketches used in the transient expression assay; (**B**) Relative Luc activity of CK and MeERF72 in tobacco leaves. Error bars represent the SD of nine technical replicates, the significant difference is assessed by ANOVA, ** *p* < 0.01.

We used the dual-luciferase reporter assay system to determine whether the MeERF72 regulates the transcription of *MeSus1 in planta*. Based on the method of Hellens et al. [14], we designed a new vector pLuc2, which integrated reporter, effector, and reference modules in the same plasmid (Figure S3B). Vectors containing either reporter only or both reporter and effector were transformed into *Agrobacterium* line LBA4404 and then transiently expressed in tobacco leaves. After detecting the relative luciferase activity in these two kinds of transiently expressed leaves, results show that the relative luciferase activity of MeERF72 was less than that of CK (Figure 7B) suggesting that MeERF72 can negatively regulate the transcript level of *MeSus1* in cassava.

## 3. Discussion

In this study, we introduced a *ccdB* gene expression cassette into pGADT7-Rec3 vector and constructed a reusable cDNA library of yeast and *E. coli* without non-recombinant, which could be easily stored and amplified for Y1H and Y2H screening. Then, a yeast one-hybrid assay was performed with the reusable cDNA library as prey and the *MeSus1* promoter as bait. Over 20 candidate TFs were screened out, and one of them is an ERF72 protein with a conserved AP2 domain and an activated domain of aa212-aa241 region. ERF72 is localized in the nucleus and could bind to the *MeSus1* promoter in vitro and in vivo, and repressed the promoter activity of *MeSus1*. These data show that ERF72 is a TF involved in plant carbohydrate fixation/starch biosynthesis.

TFs are regulators of gene expression and activate or repress the transcript levels of genes by recognizing and binding to specific cis-elements in promoters. Starch is the main component of human food, and a few TFs can regulate starch biosynthesis-related genes. In rice, OsbZIP58 binds to the promoters of multiple starch biosynthesized genes, including *AGPL3*, *Wx*, *SSIIa*, *SBE1*, *OsBEIIb*, and *ISA2* and regulates their expression levels to change starch components and other related traits [15]. Moreover, *ZmNAC36* and *ZmbZIP91* are highly expressed in maize endosperms and up-regulate the transcription of starch biosynthesis genes, such as *AGPS*, *SS*, and *ISA* and act as key regulators in maize starch synthesis [16,17]. Meanwhile, OsBP-5 (MYC) and OsEBP-89 (ERF) proteins must act synergistically, probably by heterodimerization, for the regulation of the transcription of the rice *Wx* gene [18].

Recent studies suggested that ERFs act as a key regulatory hub, integrating ethylene, abscisic acid, jasmonate, and redox signaling in the plant response to various abiotic stresses [19]. Overexpression of ERFs may improve abiotic stress tolerance in *Arabidopsis* [20], *Populus* [21,22], and rubber tree [23]. ERFs affect the biosynthesis or response of other plant hormones that regulate plant growth and development. For instance, AtERF11 promotes internodes elongation by inhibiting ethylene biosynthesis and activating gibberellin biosynthesis and signaling pathways [24], and AtERF96 positively regulates ABA responsive genes and reduced stomatal aperture in *Arabidopsis* [25]. In the present study, we found that *MeERF72* responds to several abiotic stresses and exogenous hormones. Moreover, many other cassava *ERF* genes participate in responses at low oxygen conditions and oxidative and osmotic stresses [26,27]. Furthermore, MeERF72 not only modulates the transcription level of *MeSus1*, but also contributes to abiotic stress tolerance and plant hormone signaling pathways. In the next step, we will over-express or silence *MeERF72* in cassava, then investigate the differences between wild type and transgenic plants under ethylene or other plant hormone treatments to assess the function of *MeERF72* in starch synthesis in cassava.

## 4. Materials and Methods

### 4.1. Plant Material and Treatments

Cassava cv. “KU50” was cultivated in the experimental field of the Institute of Tropical Bioscience and Biotechnology (ITBB, Haikou, China) and grown with common field management. The tissues of tip leaves, mature leaves, petioles, barks, root steles and root cortices were sampled 150 days after planting (DAP), the storage root fast developing stage, from three to five well-grown plants. Primal roots and flowers (male and female) were sampled at 60 and 240 DAP, respectively. Two storage root samples were collected 90 and 240 DAP and sliced into small cubes. All samples were immediately frozen in liquid nitrogen and stored in a −80 °C icebox until RNA extraction.

Cassava buds were sterilized by 0.1% HgCl_2_ and planted on an MS medium. Then, tissue-cultured plantlets were grown at a culture condition of 26 °C and 16/8 h in light/dark photoperiod. Approximately 45-day KU50 cultured plantlets were treated by a series of plant hormones and abiotic stresses, and their roots were sampled following the details in Table S1. All root samples were balanced mixed with 9–12 biological repeats and immediately frozen in liquid nitrogen and then stored in −80 °C icebox for the next step.

### 4.2. SMART III cDNA Library Construction

Approximately 2 mg of high-quality total RNA from storage roots of 90, 150, and 240 DAP were isolated through the sodium dodecyl sulfate (SDS) method [28] and purified following the manual of NucleoTrap^®^ mRNA kit (Macherey Nagel, Düren, Germany). Then, SMARTScribe^TM^ reverse transcriptase (TaKaRa, Tokyo, Japan) with primers of CDS III and Oligo III were used for the synthesis of a single-strand cDNA. Double-strand cDNAs were amplified by Advantage^®^ 2 Polymerase Mix with primers of 5′ PCR and 3′ PCR at optimal cycles (Table S2). Approximately 200 μL of ds-cDNA and linearized pGADT7-Rec3 vector (by *Sma* I) were co-transformed into 1.2 mL of competent cell of yeast strain Y187 through the PEG/LiAc/ssDNA method [29], and a ccdB expression module was aforehand introduced into pGADT7-Rec3 at *Sma* I site in advance (Figure S1). The co-transformed yeast cells were allowed to coat the plates of SD/-Leu, and all positive clones were scraped and combined and then concentrated to the titer of >10^8^ cfu/mL by centrifugation. Westase was used for the digestion of the concentrated yeast cells overnight, and the recombinant plasmids in the yeast were isolated through the alkaline lysis method. The isolated plasmids were transformed into competent cells of *E. coli* strain Stellar by electroporation. All the positive clones were scraped, combined, and concentrated to the titer of over 10^9^ cfu/mL. The yeast and *E. coli* cDNA library were stored under −80 °C.

### 4.3. Yeast One-Hybrid Library Screening

The 2149-bp 5′ flanking sequence of *MeSus1* (Manes.03G044400) was cloned by using the genomic DNA of cultivar KU50 as template, named *MeSus1* promoter (*MeSus1pro*). Bait vector was constructed by fusing MeSus1pro into pAbAi with *Sac* I and *Sal* I. Then, pSus1pro-AbA vector, linearized by *Bsp119* I, was integrated into the genome of yeast strain Y1HGold on the ura3-52 locus. The bait strain Y1HGold/pSus1pro-AbA survived in the medium of SD/-Ura with no more than 10 ng/mL Aureobasidin A (AbA). The plasmids of the cDNA library were transformed into competent cells of the bait strain, and the transformed yeast cells were cultured on the plates of SD/-Leu/AbA^10^ at 30 °C for 3–5 days. The plasmid DNAs of the positive yeast colonies were isolated and confirmed by PCR amplification with 5′ AD and 3′ AD primers.

### 4.4. Subcellular Location of MeERF72 Protein

For the subcellular location assay of MeERF72, the LacZ region of pCAMBIA1302 vector was removed. An enhancer of AtADH5′UTR and a newly-designed multiple cloning site (MCS) were inserted between a CaMV35S promoter and *GFP* gene, named pSL1 plus (Figure S2A). The coding sequence of *MeERF72* without the stop codon was inserted into pSL1 plus vector at the new MCS with *BamH* I and *Sal* I (35S::MeERF72-GFP). Subsequently, the vectors of 35S::MeERF72-GFP and control were introduced into onion epidermal cells through *Agrobacterium*-mediated transformation. The transformed onion epidermal cells were cultured on an MS medium in darkness at 20 °C for 36 h and then visualized by confocal microscopy.

### 4.5. DNA and Protein Interaction Confirmation in Yeast Cell

The full-length CDS and AP2-CDS (contains the AP2 domain from aa.67 to aa.166 in MeERF72 protein) of *MeERF72* were inserted into the pGADT7 vector for the infusion prey protein, AD-MeERF72 and AD-AP2, respectively. A conserved ethylene responsive element (ERE) [30], TTTGAAAT (−1384 to −1376 from ATG), located in the promoter of *MeSus1*, and a bait strain, Y1HGold/pERE-AbA, was constructed with the sequence of 5′-AACTTTCCTCTCGTTGCTTGTTTGAAATTATCATTTTCTAGTAGAATA3′ (core sequence underline). Subsequently, pGADT7-MeERF72 and pGADT7-AP2 were transformed into two bait strains Y1HGold/pERE-AbA and Y1HGold/pSus1pro-AbA, respectively, and allowed to grow in SD/-Leu/AbA^10^ medium.

### 4.6. DNA-Protein-Interaction Enzyme-Linked Immunosorbent Assay (DPI-ELISA)

AP2 was also ligated into the pCold Trigger Factor vector that had a chaperone tag in the N-terminus region (TaKaRa, Tokyo, Japan), generating pCold Trigger Factor-AP2. pCold Trigger Factor-AP2 was transformed into *E. coli* strain BL21(DE3) for protein expression. The transformed *E*. *coli* cells containing pCold Trigger Factor-AP2 were cultured in an LB liquid medium at 37 °C with 100 mg/L of Ampicillin. When the OD_600_ of the culture reached 0.4–0.8, the recombinant protein was isolated according to the method of Ma et al. [31]. The purified recombinant Trigger Factor-AP2 protein was confirmed by SDS-PAGE, and the subsequent DNA-protein interaction was confirmed by DPI-ELISA according to the method published by Brand et al. [32].

### 4.7. Transcription Activation Assay

Subclones, including N-terminus/C-terminus deletion, were fused with the GAL4 binding domain of the pGBKT7 vector and transformed into competent cells of yeast strain Y2HGold. The transformed yeast cells were doted on the medium of SD/-Trp, SD/-Trp/X-α-Gal, SD/-Trp/-Ade, and SD/-Trp/AbA^125^.

### 4.8. Expression Profile of MeERF72 By Quantitative PCR

Total RNAs were isolated through the CTAB method, and the primer pairs for quantitative PCR (qPCR) were designed by Primer-Basic Local Alignment Search Tool (BLAST) program (Table S2). qPCR assay was performed by using an Mx3000P qPCR system (Agilent, Santa Clara, CA, USA) and SYBR^®^ Premix Ex Taq^TM^ II (TaKaRa, Dalian, China), actin gene (Manes.12G150500) as reference. qPCR cycles are as follow: 90 s at 95 °C for denaturation, and 40 cycles for 10 s at 95 °C, 15 s at 58 °C, and 20 s at 72 °C for amplification. Each tested sample had four independent biological replications, and the qPCR results were analyzed by the 2^ΔΔ*C*t^ method [33] and Duncan’s test with IBM SPSS Statistics 23 software (*p* < 0.05).

### 4.9. Plant One-Hybrid

The interaction between a transcription factor (TF) and a *cis*-element or promoter *in planta* can be confirmed by plant one-hybrid through a dual-luciferase reporter assay. Hence, we designed a new vector that was derived from pCAMBIA1301 and pGreen II 0800-LUC, named pLuc2 (Figure S2B), which had three cassettes, namely, TF over-expression cassette, REN expression cassette, and Promoter-luciferase cassette. MeSus1pro was inserted into the promoter-luciferase report cassette with *Eco*R I and *Mlu* I. Then, *MeERF72* was fused to the TF overexpression cassette by *Bam*H I and *Sal* I. Subsequently, MeSus1pro::Luc and CaMV35Spro::MeERF72-MeSus1pro::Luc were separately transformed into *Agrobacterium tumefaciens* strain LBA4404. The two transformed strains were used for the infiltration of a *Nicotiana benthamiana* leaf following the protocol of Sparkes et al. [34].

## Supplementary Materials

The following are available online at http://www.mdpi.com/1422-0067/19/5/1281/s1.
