# Supplementary material for: Ethylene Responsive Factor MeERF72 Negatively Regulates Sucrose synthase 1 Gene in Cassava
Source: Int J Mol Sci. 2018 Apr 25;19(5):1281. doi: 10.3390/ijms19051281 (PMC5983797; doi:10.3390/ijms19051281)
Supplement: Supplementary file 1 [file ijms-19-01281-s001.pdf]

Supplementary

Ethylene Responsive Factor MeERF72 Negatively Regulates *Sucrose synthase 1* Gene in Cassava

Chen Liu [1,2,3,†], Xin Chen [2,3,†], Ping'an Ma [4], Shengkui Zhang [2,3,5], Changying Zeng [2,3], Xingyu Jiang [1,*] and Wenquan Wang [2,3,*]

[1] Institute of Tropical Agriculture and Forestry, Hainan University, Haikou 570228, China; liuchenneo@163.com

[2] The Institute of Tropical Bioscience and Biotechnology, Chinese Academy of Tropical Agricultural Sciences, Haikou 571101, China; chenxin@itbb.org.cn (X.C.); zsk8920@gmail.com (S.Z.); zengchangying@itbb.org.cn (C.Z.)

[3] Key Laboratory of Biology and Genetic Resources of Tropical Crops, Ministry of Agriculture, Haikou 571101, China

[4] College of Biological Engineering, Henan University of Technology, Zhengzhou 450001, China; pinganma@163.com

[5] College of Plant Science & Technology, Huazhong Agricultural University, Wuhan 430070, China

* Correspondence: jiangxingyuhu@163.com (X.Z.); wangwenquan@itbb.org.cn (W.W.)

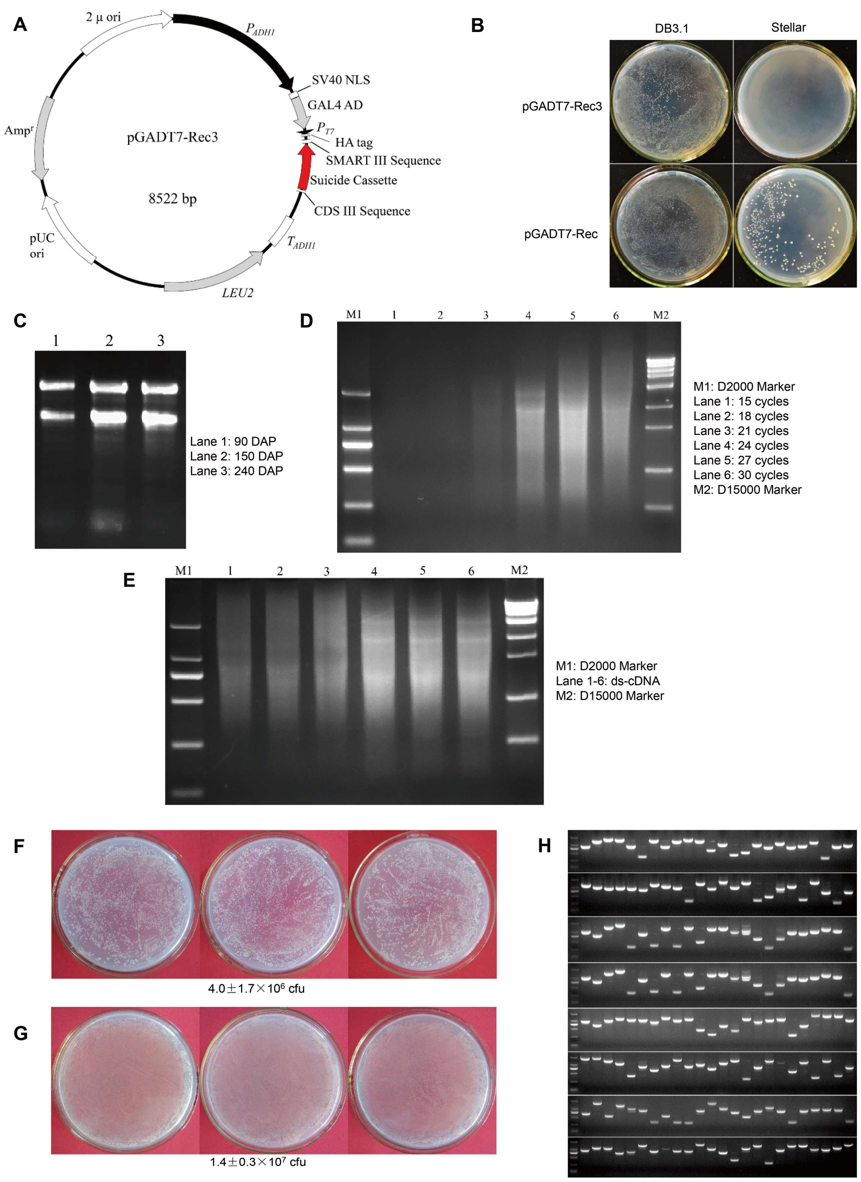

Figure S1 Flow chart of yeast one-hybrid assay. (A) pGADT7-Rec3: *ccdB* gene integrates into the pGADT7-Rec vector; (B) toxicity test results of pGADT7-Rec3 and pGADT7-Rec in DB3.1 and Stellar of *E. coli*; (C) total RNA of storage roots at three growth stages; (D) Double-strand cDNAs synthesized with different amplified cycles; (E) Synthesized double-strand cDNA with 27 amplified cycles; (F) cDNA library in yeast cells; (G) cDNA library in *E. coli* cells; (H) The size detection of insert in cDNA library.

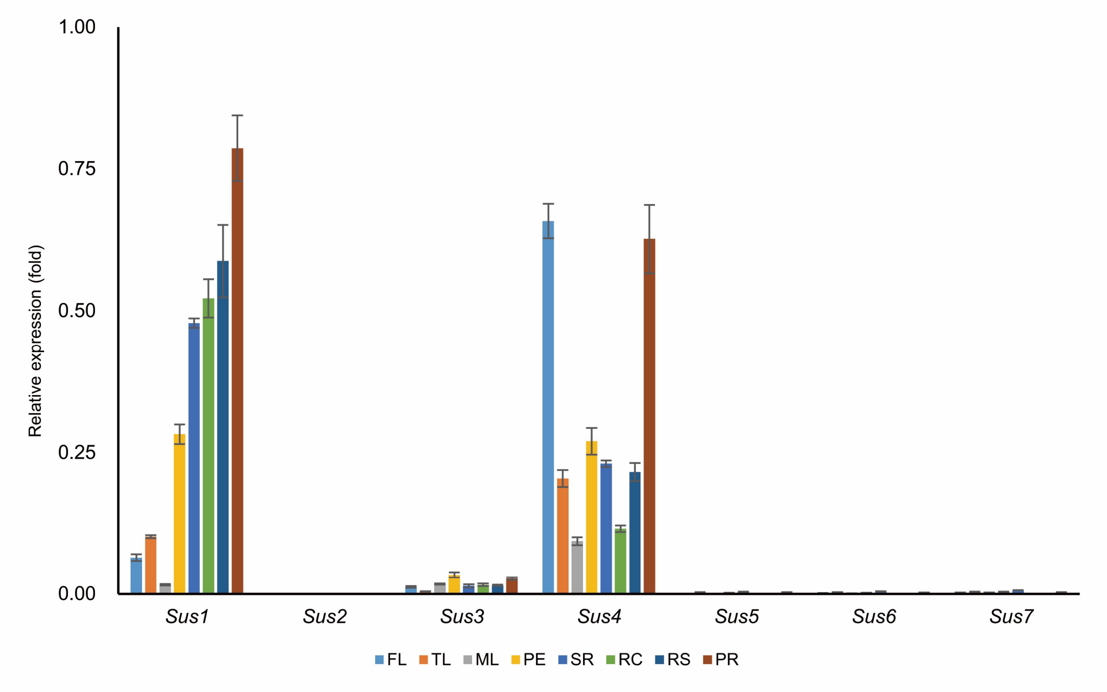

Figure S2 Transcription profile of sucrose synthase gene family in different cassava plant tissues.

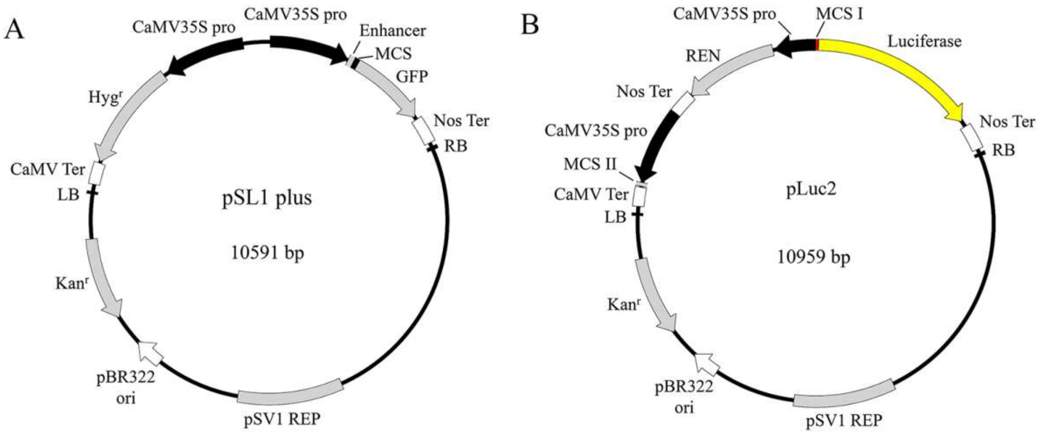

Figure S3 Sketch map of pSL1 plus vector and pLuc2 vector.

Table S1 Treatments of plant hormones and abiotic stress

Treatment	Sampling time point
Abscisic Acid (100 μM)	0 h, 1 h, 3 h, 6 h, 12 h.
Ethylene (ethephon, 10 mM)	0 h, 1 h, 3 h, 6 h, 12 h.
Gibberellin (GA_3, 100 μM)	0 h, 1 h, 3 h, 6 h, 12 h.
Salicylic Acid (5 mM)	0 h, 1 h, 3 h, 6 h, 12 h.
Auxin (IAA, 10 μM)	0 h, 1 h, 3 h, 6 h, 12 h.
Cold (4℃)	0 h, 1 h, 4 h, 12 h, 24 h.
Heat (42℃)	0 h, 1 h, 2 h, 4 h, 8 h.
Drought	0 h, 0.5 h, 1 h, 3 h, 6 h.
NaCl (200 mM)	0 h, 1 h, 3 h, 6 h, 12 h.

Table S2 Primers used in this study.

Method	Primer/Oligo Name	Primer/Oligo Sequence (5′-3′)
cDNA library construction	CDS III	ATTCTAGAGGCCGAGGCGGCCGACATG-d(T)$_{30}$VN
	Oligo III	AAGCAGTGGTATCAACGCAGAGTGGCCATTATGGCC(**GGG**)r
	5′ PCR	TTCCACCCAAGCAGTGGTATCAACGCAGAGTGG
	3′ PCR	GTATCGATGCCCACCCTCTAGAGGCCGAGGCGGCCGACA
Yeast one-hybrid	5′ AD	CTATTCGATGATGAAGATACCCCACCAAACCC
	3′ AD	GTGAACTTGCGGGGTTTTTCAGTATCTACGAT
	Sus1pro F	GAGCTCCCATTTCTTCATCCTC
	Sus1pro R	GTCGACAGCTTATGATCAGAAAAC
	ERE-N	CAACTTTCCTCTCGTTGCTTGTTTGAAATTATCATTTTCTAGTAGAATAG
	ERE-P	TCGACTATTCTACTAGAAAATGATAATTTCAAACAAGCAACGAGAGGAAAGTTGAGCT
DPI-ELISA	ERF72ad F	CATATGTGTGGTGGTGCTATTATCT
	ERF72ad R	GGATCCTTAAAAAGAAAGCTGGC
	AP2ad F	CATATGAGCCAAGTGGCCATCG
	AP2ad R	GGATCCTTAAGATGCACTTGTAGCTT
Transcription activation	ERF72 P1	CATATGTGTGGTGGTGCTATTATCTC
	ERF72 P723	GGATCCGCTTCCACTCAGC
	ERF72 P633	GGATCCGCCCAATTCTTTTTGG
	ERF72 P543	GGATCCAGAAGCGATAAGGCA
	ERF72 P283	CATATGATTTACAGAGGAATAAGGCA
	ERF72 P453	GGATCCAAAATTGAGCTTAGCC
Quantitative PCR	ERF72q F	GCCGAGGATCTCTGGTCTG
	ERF72q R	CTCAGACTTCTCACTGCCGA
	Actin F	TCTTCTCAACTGAGGAGCTGCT
	Actin R	CCTTCGTCTGGACCTTGCTG
Plant one-hybrid	ERF72p1h F	GGATCCATGTGTGGTGGTGC
	ERF72p1h R	GAGCTCAAAAGAAAGCTGGCG
